# Assessing production variability in empty and filled adeno-associated viruses by single molecule mass analyses

**DOI:** 10.1016/j.omtm.2022.11.003

**Published:** 2022-11-15

**Authors:** Eduard H.T.M. Ebberink, Alisa Ruisinger, Markus Nuebel, Marco Thomann, Albert J.R. Heck

**Affiliations:** 1 Biomolecular Mass Spectrometry and Proteomics, Bijvoet Center for Biomolecular Research and Utrecht Institute for Pharmaceutical Sciences, University of Utrecht, Padualaan 8, 3584 CH Utrecht, The Netherlands; 2 Netherlands Proteomics Center, Padualaan 8, 3584 CH Utrecht, The Netherlands; 3 Gene Therapy Technical Development Analytics, Roche Diagnostics GmbH, Nonnenwald 2, 82377 Penzberg, Germany

**Keywords:** Adeno-associated virus, AAV2, AAV8, gene-delivery vector, charge-detection mass spectrometry, mass photometry, single molecule mass analyses, empty-filled ratio, AAV biomanufacturing, native mass spectrometry

## Abstract

Adeno-associated viruses (AAVs) are useful vehicles for gene therapy because of their stability, low immunogenicity. and non-pathogenicity. However, disparity in AAV sample preparations (e.g., in capsid composition, DNA packaging, and impurities) gives rise to product heterogeneity, with possibly undesired effects on gene delivery. Ideally, AAV production should be with full control of AAV structure and genetic payload. Therefore, robust, efficient, and low material consuming methods are essential to characterize AAVs. Here, we use two emerging single-molecule techniques, mass photometry and Orbitrap-based charge-detection mass spectrometry, and show how they may efficiently and accurately characterize AAVs. We were able to resolve heterogeneous pools of particles, evaluating AAVs from two different serotypes (AAV8 and AAV2), produced by three independent production platforms, either lacking a genome or packed with a transgene. Together our data confirm that the different AAV production methods result in rather different and diverse AAV particle distributions. Especially for the packed AAVs, frequently additional subspecies were observed, next to the expected packed genome, mostly resulting from under- or overpackaging of genome material and/or residual empty particles. This work further establishes that both these single-particle techniques may become valuable tools in characterizing AAVs before they are used in gene therapy.

## Graphical abstract

**Figure:**
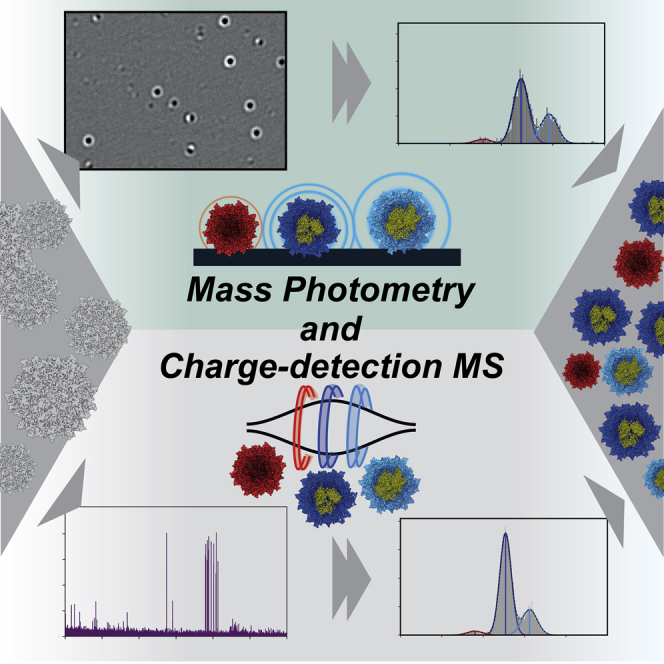

## Teaser

Two contemporary single-particle approaches, namely mass photometry and Orbitrap-based charge-detection mass spectrometry, are used to evaluate similarities and differences in empty and genome-filled adeno-associated virus particles produced by different platforms. Both techniques generally agree with each other and enable efficient monitoring of differences in both virus manufacturing and gene packaging.

## Introduction

Gene therapy is regaining momentum as a tool to battle diseases by delivery of a transgene to afflicted tissue and cells. An essential step in acquiring *in vivo* therapeutic gene expression is the safe and sustainable delivery of the genetic cargo to the targeted tissue and cells. Adeno-associated viruses (AAVs) are being predominantly investigated and used as gene-delivery vectors because of their low immunogenic response, lack of pathogenesis, and broad tropism.^1^^,^^2^^,^^3^ The AAV therapeutic potential can be illustrated by more than 200 clinical trials and 4 EMA (European Medicines Agency) and/or Food and Drug Administration-approved gene therapies.^4^

AAVs have a pseudo-icosahedral T1 capsid that contains 60 capsid protein monomers. Monomers consist of three different capsid protein isoforms (VP1, VP2, and VP3) and are encoded in a nested fashion with VP3 sharing its entire sequence with both VP2 and VP1, leaving a VP1/2 common N-terminal region, and VP2 sharing its sequences with VP1 leaving only an N-terminal VP1 unique region. The VP1 unique region is essential for cell transduction^5^; however, together with VP2 it shows to be the lowest abundant isoform in AAVs. In most reported production processes typically, a VP stoichiometry of about 5:5:50 for VP1:VP2:VP3 is observed.^6^^,^^7^^,^^8^^,^^9^^,^^10^ However, VP ratios are difficult to determine and can vary between analysis methods on the one hand and AAV production methods and batches on the other hand.^11^^,^^12^ Moreover, high-resolution techniques such as native mass spectrometry demonstrated a stochastic, expression-driven incorporation of VPs into the capsids that creates a highly heterogeneous population of capsid assemblies.^13^^,^^14^^,^^15^

In the AAV manufacturing process, transgene encapsidation can be another source of AAV disparity. Genome length and type (i.e., single-stranded or self-complementary DNA) affect its packaging process, with for instance gene truncations in case of overfilling when targeting beyond the AAV capacity.^16^^,^^17^ In addition, most AAVs remain empty during manufacturing; however, as recently demonstrated by Tran et al., they can still contain small DNA fragments.^18^^,^^19^^,^^20^ During production this can lead to a mixed set of seemingly empty, partially loaded and single-genome loaded AAVs.^12^^,^^18^^,^^19^ For clinical grade AAVs, the removal of empty or partially loaded capsids is desirable, because they are considered impurities that lack any therapeutic value but can elicit an immunogenic and potential genotoxic response.^21^ Such processes make AAV production inefficient and costly, while still bearing considerable safety concerns.^22^

Currently AAV production is predominantly performed in two host cell systems, mammalian (HEK293, HeLa) cells or insect (SF9) cells, that either by transient plasmid transfection, baculovirus infection, or stable cell lines produce the required capsid and replication proteins for AAV formation and transgene filling. Both systems produce functional AAVs with therapeutic genomes that structurally appear the same. However, the mammalian and insect-based production platforms have been reported to contain differential post-translational modification profiles, VP stoichiometries, and genome packaging efficiencies (e.g., a generally lower VP1 content is observed in insect cell-based AAVs).^20^^,^^23^^,^^24^^,^^25^^,^^26^ To characterize the process and output of manufacturing platforms, a range of analytical assays have been explored to assess yield, purity, capsid content, and AAV consistency.^19^^,^^27^^,^^28^ Unfortunately, most techniques cannot distinguish well between partially filled or single-genome containing capsids and require a relatively large amount of material and sample preparation time.^28^

Novel single-molecule-based methods that can determine molecular weights in the MDa range, such as mass photometry (MP) and charge-detection mass spectrometry (CDMS), have recently been explored to analyze AAV preparations.^29^^,^^30^^,^^31^ In MP and CDMS, AAV preparations are assessed by determining the molecular weight of individual AAV particles. MP is based on interference of light where the scattering is registered upon landing of a particle to a glass surface. Light interference due to scattering is proportionate to the mass of the particle.^32^ With recently developed mass photometers dedicated to AAV characterization, low-concentration AAV samples can be assessed.^33^ CDMS involves mass determination of ionized particles by simultaneous detection of the charge and mass-over-charge ratios of individual ions in a mass analyzer.^29^^,^^34^ Because with each scan numerous, differently charged particles can be measured, mass distributions can be acquired in a relatively short period of time (10–30 min) with minute amounts of material. This novel, single-particle mass spectrometry approach proved suitable to resolve highly heterogeneous protein assemblies such as AAVs, also on commercially available Orbitrap mass analyzers.^29^

Here we analyze by both MP and CDMS, empty and genome-filled AAV preparations from three different suppliers to probe for potential differences in the produced particles. Focusing on seemingly identical products, namely empty AAV2s and AAV8s and AAV2s and AAV8s filled with an alike transgene, our analysis allows for a direct comparison and assesses in molecular detail AAV disparity between production and purification workflows.

## Results

At present, several companies produce seemingly alike AAV particles, often with seemingly alike packed transgenes. However, due to the use of different host cells, production, and purification processes, these products may actually still be rather different. To provide a representative panel of seemingly alike AAV products, we analyzed samples linked to two AAV serotypes, AAV2 and AAV8, either lacking or containing a CMV-GFP encoding genome. AAVs were obtained from three different suppliers, who produced them either by using insect cell- (Virovek) or mammalian cell-based platforms (Sirion and Vigene). For nomenclature of the different samples, the vendor names are abbreviated throughout this work (i.e., Virovek = Vir, Sirion = Sir, and Vigene = Vig). Below we describe our findings per serotype and applied single-particle analysis method.

### Characterization of empty and filled AAV8 by MP

**Figure 1:**
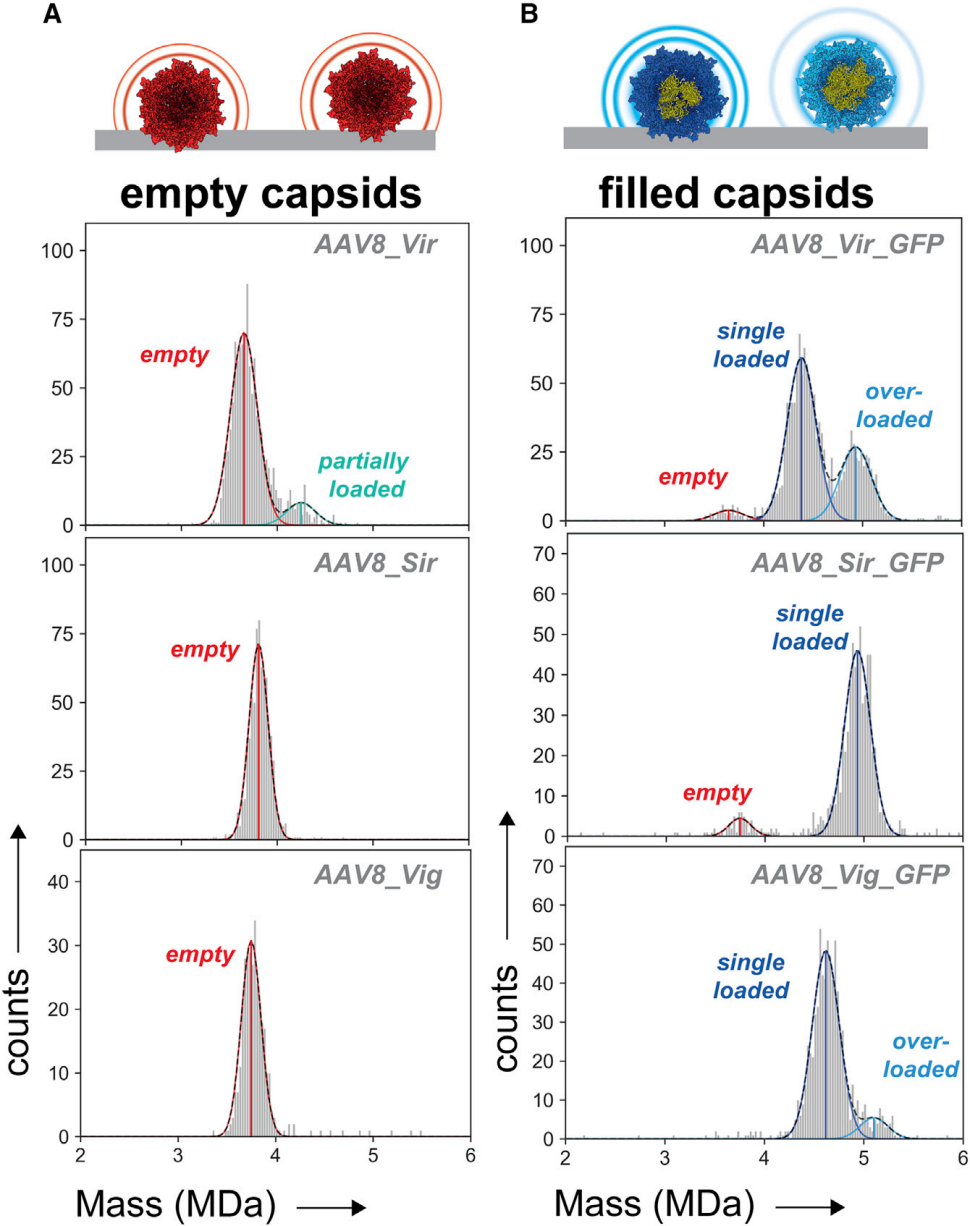
Mass photometry analyses of empty and ssDNA packaged AAV8 capsids obtained from three different suppliers (A) Mass histograms of supposedly empty AAV8 capsids. The scattering event of each particle landing on the glass surface is translated into a particle mass and is classed in bins containing a bin width of 25 kDa. (B) Mass histograms of supposedly filled AAV8 capsids following production in the presence of a CMV-GFP transgene. Likewise, to the empty capsids, mass histograms were constructed with bin widths of 25 kDa. For each AAV8 sample, a single, representative mass histogram is displayed. For the most abundant species, Gaussian distributions were fitted and the mean masses are displayed as vertical lines. The average fitting over at least three mass distributions is given in Figure S3. Vir = Virovek; Sir = Sirion; Vig = Vigene; red = empty capsids; dark blue = single genome loaded capsids; light blue = overloaded capsids; turquoise = partially loaded capsids.

The purity and sample homogeneity of AAV capsid preparations were first assessed by mass photometry (Refeyn Samux MP). For this purpose, the supplied AAV8 stock solutions were diluted in PBS and several hundreds of scattering events were acquired by MP (Figure S1). Following processing and calibration, the obtained masses were plotted in mass histograms. The mass distributions mostly displayed single populations of empty AAVs (Figure 1A). Gaussian fits of the mass distributions were centered around 3.66 ± 0.05, 3.80 ± 0.02, and 3.74 ± 0.03 MDa for empty capsids of AAV8_Vir, AAV8_Sir, and AAV8_Vig, respectively (Table 1). Variability in the centered masses hint at differences in VP built-up and/or VP post-translational processing, to be expected from AAVs originating from different production platforms. Only in AAV8_Vir a small side population of capsids exhibits a higher mass. AAV8_Vir also displayed a substantial broader peak width with a full-width-at-half-maximum (FWHM) of 0.27 ± 0.02 compared with 0.24 ± 0.01 and 0.23 ± 0.01 of AAV8_Sir and AAV8_Vig, respectively (Figure 1A and Table 1). The AAV8_Vir pool of (empty) AAVs thus displays more mass variation and subspecies, either by unintentional packing of DNA or incomplete purification of the empty capsids.

**Table 1:** Overview of the fitted means and full-width-at-half-maximum values of AAV subspecies as determined by MP and CDMS

	AAV8 capsids			AAV2 capsids		
	*Theoretical MW (VP ratio 5:5:50) = 3.73*			*Theoretical MW (VP ratio 5:5:50) = 3.74*		
	*Empty*	*Partially or single loaded*	*Overloaded*	*Empty*	*Partially or single loaded*	*Single loaded or overloaded*
**MP Virovek**						
Mean	3.66 ± 0.05	4.04 ± 0. 05		3.71 ± 0.01		
*FWHM*	0.27 ± 0.02	0.55 ± 0.07		0.34 ± 0.07		
**CDMS Virovek**						
Mean	3.72 ± 0.02	4.12 ± 0.03		3.75 ± 0.02		
*FWHM*	0.26 ± 0.01	0.50 ± 0.05		0.32 ± 0.02		
**MP Virovek_GFP**						
Mean	3.69 ± 0.01	4.42 ± 0.03	4.97 ± 0.03		4.37 ± 0.07	4.94 ± 0.08
*FWHM*	0.60 ± 0.10	0.35 ± 0.03	0.43 ± 0.1		0.42 ± 0.06	0.38 ± 0.04
**CDMS Virovek_GFP**						
Mean	3.83 ± 0.02	4.56 ± 0.01	5.12 ± 0.01		4.53 ± 0.04	5.16 ± 0.04
*FWHM*	0.46 ± 0.01	0.32 ± 0.01	0.48 ± 0.03		0.51 ± 0.05	0.37 ± 0.05
**MP Sirion**						
Mean	3.80 ± 0.02			3.88 ± 0.04		
*FWHM*	0.24 ± 0.01			0.28 ± 0.05		
**CDMS Sirion**						
Mean	3.82 ± 0.01			3.82 ± 0.02		
*FWHM*	0.23 ± 0.002			0.28 ± 0.03		
**MP Sirion_GFP**						
Mean	3.74 ± 0.03	4.90 ± 0.04		3.94 ± 0.05	4.60 ± 0.06	5.13 ± 0.07
*FWHM*	0.35 ± 0.08	0.35 ± 0.05		0.37 ± 0.07	0.48 ± 0.17	0.34 ± 0.04
**CDMS Sirion_GFP**						
Mean	3.83 ± 0.02	5.00 ± 0.02		3.81 ± 0.02	4.41 ± 0.02	4.96 ± 0.02
*FWHM*	0.34 ± 0.02	0.34 ± 0.01		0.27 ± 0.01	0.47 ± 0.01	0.36 ± 0.003
**MP Vigene**						
Mean	3.74 ± 0.03			3.79 ± 0.03		
*FWHM*	0.23 ± 0.01			0.22 ± 0.01		
**CDMS Vigene**						
Mean	3.78 ± 0.004			3.77 ± 0.004		
*FWHM*	0.22 ± 0.003			0.29 ± 0.03		
**MP Vigene_GFP**						
Mean		4.63 ± 0.06	5.12 ± 0.05	3.83 ± 0.06	4.55 ± 0.07	
*FWHM*		0.35 ± 0.02	0.42 ± 0.06	0.35 ± 0.02	0.40 ± 0.05	
**CDMS Vigene_GFP**						
Mean		4.57 ± 0.01	5.05 ± 0.01	3.82 ± 0.01	4.56 ± 0.01	
*FWHM*		0.37 ± 0.01	0.27 ± 0.02	0.33 ± 0.002	0.50 ± 0.01	

**Figure 2:**
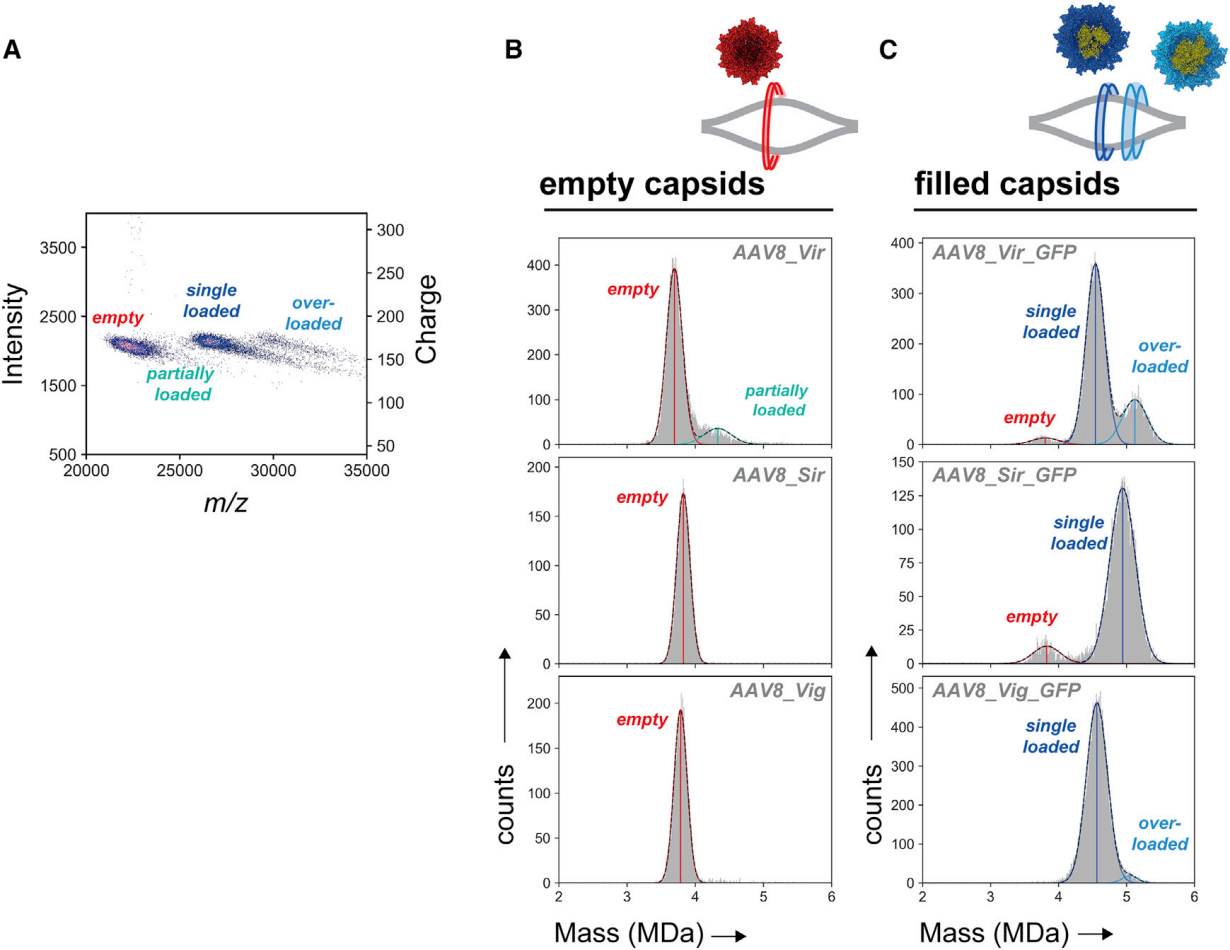
Orbitrap-based charge-detection mass spectrometry on empty and ssDNA packaged AAV8 capsids AAV8 capsids were electrosprayed into a UHMR Orbitrap analyzer to detect the charge and mass-over-charge ratio. (A) Displayed is an overlay of the 2D-histograms of *m/z* versus intensity and charge from AAV8_Vir and AAV8_Vir_GFP. Bin widths of 25 Th and 10 arbitrary units were used for, respectively, *m/z* and intensity. The color code represents the number of particles in each bin ranging from blue to red for respectively low and high counts. (B) The masses of empty capsids were calculated from the 2D-histogram and plotted in a 1D mass histogram with a bin width of 10 kDa. (C) Filled AAV8 capsids were processed the same way as empty capsids. Different species of AAVs can be distinguished containing different amounts of added mass in both the mass histograms as well as in the z-space 2D histograms. Vertical lines are drawn at the fitted mean. The average fitting over at least three independent measurements is given in Figure S3. Vir = Virovek; Sir = Sirion; Vig = Vigene; red = empty capsids; dark blue = single genome loaded capsids; light blue = overloaded capsids; turquoise = partially loaded capsids.

**Figure 3:**
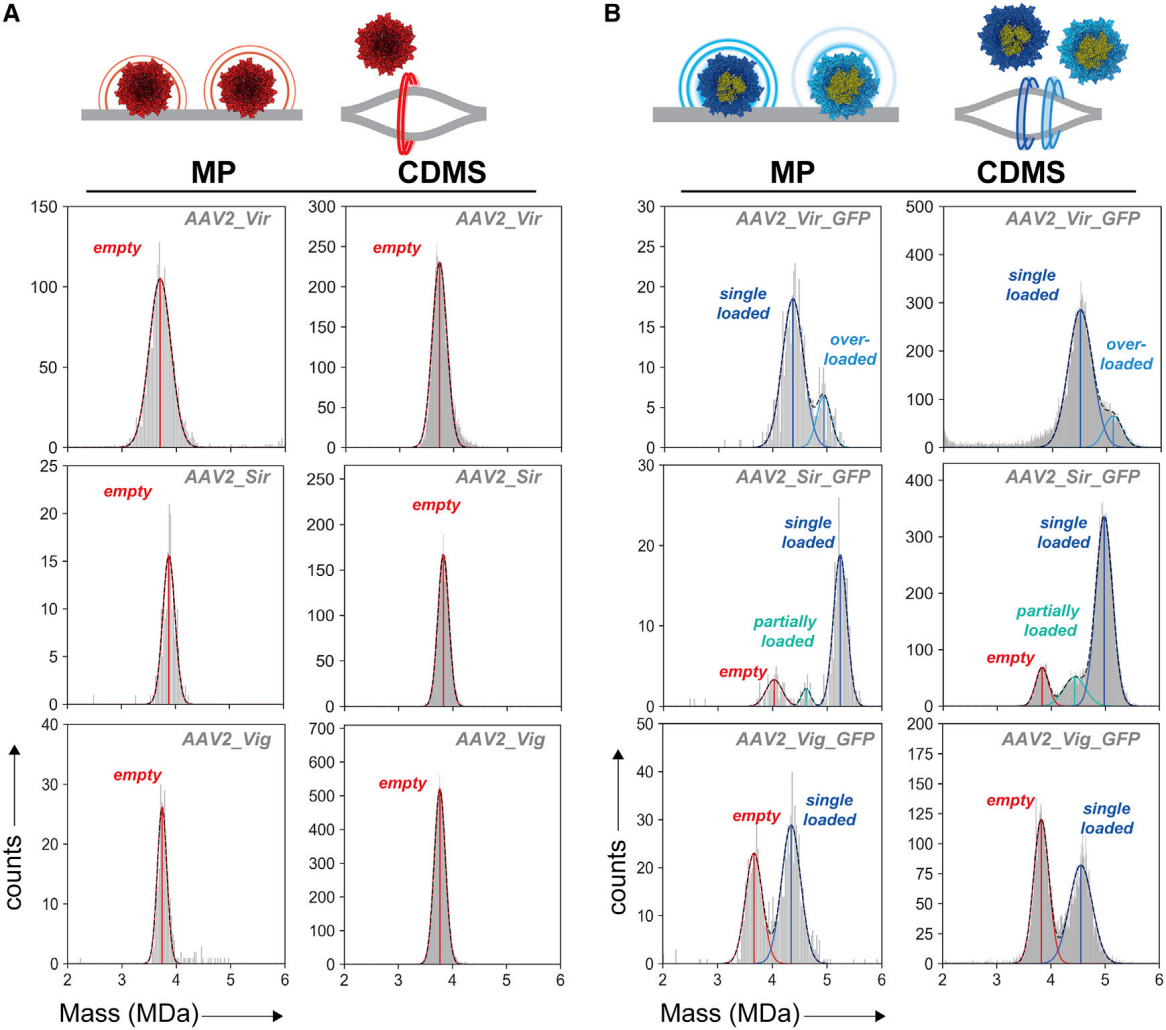
Mass photometry and charge-detection mass spectrometry on empty and ssDNA packaged AAV2 capsids (A) Constructed mass histograms of empty AAV2 capsids measured by MP and CDMS. (B) Filled AAV2 capsids contained a CMV-GFP transgene. Displayed are representative mass histograms as obtained by MP and CDMS. Mass histograms following MP and CDMS were plotted with, respectively, 25 kDa and 10 kDa bin widths. Vertical lines are drawn at the fitted mean. The average fitting over at least three measurements is given in Figure S3. Vir = Virovek; Sir = Sirion; Vig = Vigene; red = empty capsids; dark blue = single genome loaded capsids; light blue = overloaded capsids; turquoise = partially loaded capsids.

Mass photometry (MP) and charge-detection mass spectrometry (CDMS) acquired mass distributions from at least three independent measurements. Mass distributions were fitted with a Gaussian function for every subspecies (empty, partially loaded, single loaded, or overloaded particles) as seen in Figure 1, Figure 2, Figure 3. Taking together the Gaussian fits of all repeats, the overall mean and full-width-at-half-maximum (FWHM) value with accompanied standard deviation is given below. Traces of the averaged fit of each adeno-associated virus (AAV) sample is given in the supplemental information, Figure S3.

Values in this table represent fitted mean ± standard deviation or FWHM ± standard deviation.

For all these empty AAV8 samples, we also acquired alike samples with a packaged CMV-GFP transgene. Already at first glance, the particles display mass distributions that are highly divergent between the different manufacturers, while these packaged AAV8s essentially express the same protein (Figure 1B). Taking into account that per supplier the size and design of genomes differs substantially (Table S1), we could readily assign single-genome packed AAVs for the most abundant peaks. The AAV8_Sir_GFP displayed the most abundant mass distribution around 4.90 ± 0.04 MDa, clearly revealing that it has packed a substantially larger genome (Figure 1B, Table 1 and Table S2). For the AAV8_Vir_GFP and AAV8_Vig_GFP, the most abundant particle distribution exhibited added masses of respectively 0.73 to 0.89 MDa compared with their empty capsid equivalents (Figure 1B, Table 1, and Table S2). This falls in the range of packaging a single genome (Table S1). For both samples, however, a smaller side population is present in both preparations that displayed an extra mass of ∼0.5–0.6 MDa. This seems to fall short for packaging an extra, complete genome but instead demonstrates partial overfilling of the capsids. Notably, throughout this sample set, the total incorporated “added mass” does not extend beyond 1.4 MDa (∼4.6 kb single-stranded DNA [ssDNA]) (see Table S2 for an overview of the measured mass differences).

### Characterization of empty and filled AAV8 by CDMS

Although MP has the advantage of speed and ease of analysis, it has been noted that exact molecular weights for non-conventional viral samples can be error-prone, because for MP a universal contrast-to-mass conversion is lacking.^31^^,^^35^ To validate the MP data, the same set of samples were in parallel subjected to CDMS measurements on a UHMR Orbitrap analyzer (Figure 2). For this purpose, AAVs were electrosprayed and measured in the Orbitrap taking individual *m/z* and charge values.^36^ Upon classification of the particles in a 2D histogram plot over *m/z* and charge, AAV subspecies can be distinguished (Figures 2A and S2). As shown previously, with a 512-ms transient and an *m/z* of about 25,000 for the AAVs, the charge uncertainty in our measurements is expected to be about 3.5 charges.^36^ Subsequent conversion to mass and plotting into a 1D histogram also displays the different AAV subspecies (Figures 2B and 2C and Table 1). As observed by MP, the AAV8_Vir sample exhibits a broader peak width and contains an extra small subpopulation of heavier particles (Figure 2B). The empty capsids of AAV8_Vig and AAV8_Sir displayed a single, monodisperse Gaussian-like distribution. Also here, the fitted means deviate from each other in a similar fashion as seen by MP (AAV8_Sir > AAV8_Vig > AAV8_Vir), again hinting at distinct VP ratios and/or post-translationally processing.

When measuring genome-packed AAV8s, the CDMS mass histograms resemble those seen earlier with MP, displaying the same distribution of AAV8 subspecies (Figure 1, Figure 2B, 2C, and S3). Overall, the mass distributions extracted from the CDMS data appear to be highly similar to those obtained by MP. The similarity in mass distributions containing a high number of particles demonstrates that, as seen before, (packaged) AAVs are stable and well-suited for CDMS under the current settings (see materials and methods) in the gas phase.^29^ Notably, genome-packed AAVs tend to have a broader spread in charges following some charge reduction. The FWHM of these distributions is in the same range between MP and CDMS, for empty as well as genome-containing capsids. Moreover, the mass differences between distributions are nearly the same in MP and CDMS measurements (Table S2). That the MP fitted means slightly differ from CDMS (and not the transgene mass) is likely due to an offset created by the pure protein based MP calibration (i.e., low molecular weight thyroglobulin multimers) that lacks an ssDNA component.

### Mass determination of empty and filled particles of the AAV2 serotype

Besides AAV8, MP and CDMS can readily be applied to AAVs of different serotype and/or design. The capsids of AAV2 and AAV8 serotypes share a high sequence identity of 82% and comparable tropism, although AAV2 appears to be less stable compared with AAV8.^3^^,^^37^^,^^38^ From the same manufacturers, AAV2 capsids were examined with or without a CMV-GFP transgene. The AAV2 mass distributions extracted from MP and CDMS are displayed in a side-by-side manner and, as seen for AAV8, reveal a striking similarity regardless of the assay used (Figures 3 and S3). Empty capsids show a monodisperse population of masses. Contrary to AAV8_Vir, we cannot detect any AAV2s with added mass that would indicate unwanted, coincidental packaging.

Translocation of the genome into the AAV2 capsids displayed more variety (Figure 3B). In AAV2_Vir_GFP, again a second population was observed with additional mass beyond what one would expect based on packaging of a single genome. AAV2_Sir_GFP shows a small additional mass distribution that corresponds to empty capsids. For the same sample, there is also a subpopulation of AAVs with an additional ∼0.6–0.7 MDa. This observed mass shift is insufficient for packing an extra intact single genome (Table S1). The partial filling of capsids has been described before; however, it is the first time we observe partial filling so clearly here.^12^^,^^29^^,^^30^ Despite these small subpopulations, the most abundant species corresponds with packaging of a single, intact genome (1.15–1.36 MDa) (Table S1). When we look at the derived genome mass (filled particles – empty particles) for both AAV8_Sir_GFP and AAV2_Sir_GFP, it is slightly above the theoretical value (Tables S1 and S2). This can be explained by packing of counterions in the ssDNA as observed before for genomes in the 1 MDa range.^30^ In both AAV2_Sir_GFP and AAV2_Vir_GFP, the most abundant species has a mass that suggests that a single genome is taken up. Only AAV2_Vig_GFP has a substantial part of the AAVs that seems empty based on the mass distribution (Figure 3B). Overall, the filled AAVs of serotype 2 contain more variability in packaging compared with AAV8.

### VP ratios and VP PTM profiling in the different AAV constructs and serotypes

When investigating the capsids by MP and CDMS we could observe substantial differences in AAV molecular weights compared with the theoretical mass based on the generally assumed VP stoichiometry of 5:5:50 in the 60-mer capsid (Table 1). Therefore, we performed capillary electrophoresis-sodium dodecyl sulfate (CE-SDS) in combination with liquid chromatograph-mass spectrometry (LC-MS) to determine the VP1:VP2:VP3 ratios and PTM profiles of all samples. The CE-SDS revealed that the VP content does not always follow this 5:5:50 stoichiometry (Table 2). Especially for AAV_Sir and AAV_Vig, relatively more copies of VP1 and VP2 are incorporated into the capsids, leading to average stoichiometries closer to 8:12:40. When calculating the theoretical values based on these determined VP ratios, fitted means of the CDMS and MP followed the molecular weights more closely (Table 1, Table 2 and 2). The presence of a transgene did not substantially affect the measured VP ratios (Table 2). Also, the difference in serotype, AAV2 or AAV8, did not influence the VP ratio as much when compared with differences observed between AAV production platforms.

**Table 2:** Viral protein stoichiometry of the AAV particles

	VP1: VP2: VP3	VP ratio/PTM adjusted theoretical mass (MDa)
**AAV8**		
AAV8_Vir	4.0 5.4 50.6	3.71
AAV8_Vig	7.6 12.0 40.4	3.83
AAV8_Sir	8.2 14.5 37.3	3.86
AAV8_Vir_GFP	4.8 3.9 51.3	3.72
AAV8_Vig_GFP	8.6 11.9 39.6	3.85
AAV8_Sir_GFP	7.8 13.9 38.3	3.85
**AAV2**		
AAV2_Vir	6.2 4.4 49.4	3.76
AAV2_Vig	6.4 8.6 45.0	3.79
AAV2_Sir	8.5 12.9 38.5	3.87
AAV2_Vir_GFP	4.1 4.5 51.4	3.72
AAV2_Vig_GFP	7.5 9.4 43.0	3.82
AAV2_Sir_GFP	8.4 12.9 38.7	3.86

Average VP1/VP2/VP3 stoichiometry of the capsids as obtained by CE-SDS of AAV8 and AAV2 samples from the three different suppliers. These stoichiometries were normalized to the total number of subunits, n = 60. According to the VP stoichiometry, as determined by capillary electrophoresis-sodium dodecyl sulfate (CE-SDS), the theoretical average mass of the resulting AAV capsids were calculated for each AAV supplier/production run. Sir = Sirion; Vig = Vigene; Vir = Virovek.

**Figure 4:**
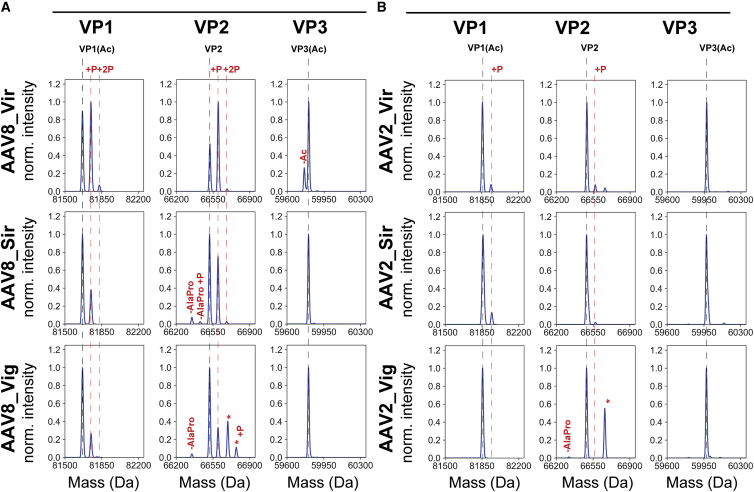
PTM profiling of viral protein subunits VP1, VP2, and VP3 derived from the different empty AAV8 and AAV2 samples Displayed are the deconvoluted masses of VP1, VP2, and VP3 following LC-MS on an Exploris 480 Orbitrap mass analyzer in intact protein mode of (A) AAV8 and (B) AAV2. Indicated with a black dashed line is the most abundant VP proteoform across the three sample sets (i.e., AAV_Vir, AAV_Sir, AAV_Vig). Major PTMs (i.e., phosphorylation, acetylation) are indicated with a red dashed line. Smaller PTMs are separately indicated in the individual plots. Indicated with an asterisk are VP2s that contain an unidentified modification of approximately +172 Da. The intensities of the deconvoluted masses are normalized to the most abundant peak in the plot. See Tables S3 and S4 for all measured masses and assignments.

Following denaturing of the empty AAV particles, PTM analysis following LC-MS of the intact VP proteins indicated highly abundant modifications in the AAV8 VPs (Figure 4A, Table S3, and Figure S4). The most dominant PTM, phosphorylation of the VP1 and VP2, is highly abundant for especially AAV8_Vir compared with AAV8_Sir and AAV8_Vig (Figure 4A). Small N-terminal truncations of VP2 with loss of the initiating Alanine and Proline residues are present in AAV8_Sir and AAV8_Vig while absent in AAV8_Vir. As expected, acetylation of VP1 and VP3 was ubiquitous in both AAV serotypes and across suppliers. VP2 remained devoid of N-terminal acetylation. A small fraction of non-acetylated VP3 could also be detected in AAV8_Vir (Figure 4A). The PTM pattern of AAV2 appears different and more restrained compared with AAV8. To illustrate, the AAV2 samples show much less phosphorylation (Figure 4B and Table S4). The observed N-terminal truncations in AAV8_Sir are absent in its AAV2 counterpart. Only AAV2_Vig shows a minor amount of VP2 that lacks the N-terminal Alanine and Proline residues. Solely in AAV8/2_Vig, VP2 has modifications with a mass shift of about +172 Da, which cannot readily be annotated (indicated with asterisk). Of note, this modified VP2 retains the same phosphorylation profile as regular VP2 (Figure 4). In summary, the amount and type of modifications are dissimilar between manufacturers and are more prevalent in the AAV8 samples. This in contrast to AAV2, which seems less prone to PTMs.

Several studies of intact and digested VPs reported the presence of glycosylation sites and phosphorylation of VP3.^8^^,^^23^^,^^39^^,^^40^ In contrast, in our assay we did not detect any appreciable phosphorylation of VP3 or addition of glycan moieties to AAV2 or AAV8. This does not exclude such modifications, but they would be relatively low abundant compared with the predominant modifications we observe (phosphorylation of VP1 and VP2 and acetylation of VP1 and VP3). Recently, a smaller VP3 variant has been described that is transcribed at a second ribosomal initiation site at Met211 in AAV2 and Met212 in AAV8.^11^ The novel Alanine N-terminus of this VP3 variant is also acetylated, which results in VP proteins of about 59,192 kDa for AAV8 and 59,301 kDa for AAV2. In our data for both serotypes, when analyzing the deconvoluted spectra, this VP3 variant was also detected (Figure S5), albeit the VP3 variant was of relatively minor abundance.

## Discussion

AAVs have become an indispensable vector system within the field of gene therapy. AAV production for pharmaceutical purposes requires upscaling and tight control on the quality and consistency of the products. However, it remains difficult to reliably produce consistent AAVs and by extension determine the quality and safety of (packaged) AAVs. Here we used two novel single-molecule techniques to investigate and characterize the AAV heterogeneity and quality. For all the interrogated samples, both MP and CDMS displayed highly similar distribution patterns (Figure 1, Figure 2, Figure 3). The mass uncertainty for both techniques is well below 2%. This is in line with earlier work where we used MP and CDMS on macromolecules of mixed nucleic acid/protein (ribosomal) content.^35^ MP has the advantage of AAV detection under physiological conditions in a straightforward, non-laborious way. Unfortunately, it lacks the same mass accuracy as CDMS mostly due to the absence of an adequate AAV mass range calibrant. CDMS has an accurate charge-based calibration and therefore better intrinsic mass accuracy, although mass spectrometry requires harsher measuring conditions (e.g., buffer exchange, electrospray, high voltage transmission, low pressure) with awareness of potential bias.^29^^,^^35^ In this study, the acquired values by MP and CDMS support each other with highly similar peak abundances and masses for all AAV subpopulations. In addition, the packaged mass of AAVs inferred by mass subtraction are near equal (Table S2). This demonstrates that MP and CDMS are excellent workflows for evaluation of AAV post-production composition as well as DNA packaging.

We assessed AAV pools of three different vendors of which one (i.e., Virovek) uses the SF9 insect cell platform for AAV production.^41^ We observed distinct features of these AAVs compared with the human cell line-based AAVs. Most notably, when investigating the capsid VPs, insect cell AAVs are different in both stoichiometry and post-translational processing (Table 2 and Figure 4).^25^ Next to bulk assay, also MP and CDMS on both insect-based empty AAV8 and AAV2 are different and tend to show broader mass distributions (Figure 1, Figure 2, Figure 3A, 2B, and 3A). Presumably empty, this cannot be attributed to differential packaging of a transgene. Why particular insect AAVs display such differences remains elusive. Perhaps, the insect cell post-translational machinery is intrinsically different or, alternatively, the high AAV productivity and speed in insect cells can bring forth differences in VP stoichiometry, PTMs, and/or unintentional packaging of DNA fragments or host cell proteins.^20^^,^^23^ That unraveling the source of insect composition variability is important can be seen in the co-administration of empty insect cell AAVs, which, in contrast to human AAVs, inhibits transduction.^23^ The insect cell-based AAV platform has several advantages such as a high production yield without the use of serum or helper plasmids.^18^ Therefore, a better understanding of the insect cell-driven structural variability and its influence on efficacy and safety remains indispensable.^42^

One particular PTM that stood out, for especially insect cell AAVs, was the phosphorylation of VP1 and VP2 (Figure 4A). A similar pattern of phosphorylation was not observed in AAV2, which points toward an AAV8 specific phosphorylation site within the VP1/VP2 common region (Figure 4B). One serine residue that fits these conditions and has been described before as being phosphorylated is Ser153.^23^ It is generally perceived that before intracellular processing, the VP1/VP2 common region remains internal of the capsids.^43^ With substantial phosphorylation described here, it is worthwhile to interrogate the phosphorylated VP1/VP2 common region and probe a specific interior function of phosphorylation (e.g., in packaging or release of DNA). Otherwise, phosphorylated Ser153 can influence the downstream transduction and transcription process once it is externalized, as seen for alanine substitutions of nearby serines (Ser155-Ser157) in AAV2.^44^

Besides the capsid structure, internalization of ssDNA is a second source of inconsistency in AAV composition.^20^ In the presence of a packageable transgene not just a dual set of species, either completely empty or single-genome filled capsids, are generated. Particles with variable less or extra packaging are overtly present (Figure 1, Figure 2, Figure 3). By using CDMS and MP we can resolve the variable filled populations that are usually disregarded in assays that distinguish only between empty or filled capsids (electron microscopy, UV detection, dynamic light scattering). In previous CDMS experiments, packaging of extra material beyond a single genome has been described but in lower abundancies.^12^^,^^29^ In this work, we observe an obvious amount of AAVs with “overpackaging,” in particular for the insect cell-derived AAV8 and AAV2 (Figure 1, Figure 2, Figure 3B, 2C, and 3B). Strikingly, in case of encapsidation of extra material, AAV masses stay centered close to the packaging limit of 1.6 MDa and do not exceed it.^16^ In addition, the DNA containing vectors appear more heterogeneous, as the mass distribution widths are higher compared with empty AAVs (Figure 1, Figure 2, Figure 3). Most likely, this is attributed to small truncations or elongations of single ssDNA chains, collateral packaging of small DNA fragments, or DNA shielding counterions.^20^^,^^25^^,^^30^ With such variable and heterogeneous DNA uptake being the rule rather than the exception, MP and CDMS are well-suited techniques for quick assessment of DNA packaging.

In characterizing the samples presented here, seemingly identical (empty) AAV capsids are in fact highly diverse in mass. Once a transgene is targeted to the capsids, an extra source of variability is added. This work evidently exposes the divergent mass distributions and different subspecies that result from the many underlying variables in the AAV production process. Factors that are either biology-related attributes such as serotype, type of host cell, and expression levels or purely manufacturing-process related factors that depend on the production media and conditions (adherent versus suspension), size, and design of genome, as well as the purification methods used. At the moment, there is only very limited knowledge about the influence each factor has and thus AAV manufacturing creates, next to the desired therapeutic product, unwanted side-products. MP and CDMS are ideal techniques to efficiently assess the amount and nature of potentially harmful unwanted side-products. Co-analysis of VP ratios and VP modification by whole VP LC-MS may help to annotate the measured masses of the particles and/or explain the observed mass heterogeneities. Therefore, in addition to existing analytical assays, intact AAV mass analyses by MP and/or CDMS is becoming an essential part of the therapeutic AAV biomanufacturing and extended characterization.

## Materials and methods

### MP

In preparation of the MP measurements, stock solutions of AAVs (ranging from 0.5 × 10^13^ to 5 × 10^13^ Vp/mL) were pre-diluted in PBS (Gibco) to about 0.5 × 10^10^ to 2 × 10^11^ Vp/mL. Clean microscope coverslips (24 mm × 50 mm; Paul Marienfeld GmbH) were acquired by serial rinsing with Milli-Q water and HPLC-grade isopropanol (Fisher Scientific Ltd.). CultureWell gaskets (Grace Biolabs) were placed as container wells for the AAV dilutions. Coverslips were mounted on a Samux mass photometer (Refeyn Ltd.) and 12 μL of PBS buffer was used to set the focus. For each measurement, 3 μL of AAV solution was applied and mixed in the well. Movies were recorded for 60 or 120 s at 100 fps. Contrast-to-mass conversion was done by measuring a thyroglobulin multimer mix (Sigma, T9145). Three contrast rates were aligned with masses of 335, 670, and 1,340 kDa in a calibration curve. MP data were processed using DiscoverMP (Refeyn Ltd.), following export of the data mass histograms, and Gaussian fits were acquired using SciPy and in-house Python scripts.^45^

### CDMS

Prior to CDMS measurements AAV stock solutions (ranging from 0.5 × 10^13^ to 5 × 10^13^ Vp/mL) were buffer exchanged to 75 mM ammonium acetate. About 30 μL of stock solution was diluted into 450 μL of 75 mM ammonium acetate and concentrated to 20 to 30 μL using a 50K MWCO filter (Merck Millipore) by centrifugation for 10 min at 6,000 × *g*. This step was repeated an additional five times. Alternatively, about 30 μL of stock solution was buffer exchanged using a 40-kDa MW limit Bio-Rad P-30 Micro Bio-Spin column, following vendor recommendations. About 3 μL of buffered exchanged AAVs was loaded into a gold-coated borosilicate capillary (prepared in-house) for nanoelectrospray ionization. AAVs were measured on an Orbitrap Q Exactive UHMR mass spectrometer (Thermo Fisher Scientific) in positive mode. The *m/z* calibration of instrument was performed using cesium iodide clusters in the range between 350 and 12,000 *m/z*. For the CDMS measurements of AAVs, an *m/z* range between 10,000 and 40,000 was used with a resolution of 100,000 at 400 m/z (512 ms ion transient). The noise level threshold was fixed at 0. The in-source-trapping desolvation voltage was set between −75 V and −150 V, and an HCD voltage of 100 to 175 V was used for maximal ion transmission. In-source trapping was allowed for 4 ms with 4 V trapping voltage. Pressure settings ranged from 2 to 3 (UHV between 1.5 and 5 × 10^−10^ mbar) and Xenon was used as the collision gas.^29^ After multiscan acquisition for 10 to 30 min, RAW files were centroided and converted into mzXML format for further processing by removal of dephased ions.^35^^,^^36^ The mzXML files are deposited in the MassIVE repository (ftp://massive.ucsd.edu/MSV000090582/). Ion intensities were normalized to 1 s sample injection time and a calibration factor of 12.521 (normalized arbitrary intensities/charges) was used to convert intensity to charge. According to the determined charge state, a resulting formula m = *m/z* ∗ z − z was used to calculate the mass of each single ion in kDa. Histograms of the calculated masses were plotted and Gaussian fits were applied to the distinctive subpopulations.

### LC-MS of intact VP1, VP2, and VP3

AAV particles were denatured by addition of 2% formic acid. About 1 to 5 μg of acidified VPs were separated on a Vanquish Flex UHPLC (Thermo Scientific) equipped with a MAbPac column (1 mm × 100 mm) (Thermo Scientific) for reversed-phase separation incubated at 80°C. The liquid chromatography gradient was set from 71% mobile phases A (water/0.1% trifluoroacetic acid) and 29% B (ACN/0.1% trifluoroacetic acid) to 65% A and 35% B over 14 min. A flowrate of 150 μL/min was used and eluted proteins were sprayed into an Exploris 480 Orbitrap mass spectrometer (Thermo Scientific). MS data were collected with the instrument set to intact protein mode and low-pressure setting. The Orbitrap resolution parameter was set to 7,500 (at 200 m/z) corresponding to a 16-ms transient signal. Full MS scans were acquired for the range of 500 to 4,000 m/z with the automatic gain control target set to 300%. The maximum injection time was defined at 50 ms with 5 μscans recorded. Spray voltage was set at 3.5 kV, capillary temperature 350°C and probe heater temperature 100°C. Sheath and Aux gasses were set at 15 and 5 respectively. Deconvolution of the masses retrieved from the RAW files were done using BioPharmaFinder 3.2 (Thermo Scientific). Deconvolution was performed using the ReSpect algorithm between 3 and 15 min using 0.1 min sliding window with 25% offset and a merge tolerance of 30 ppm, with noise rejection set at 95%. The output mass range was set at 5,000 to 100,000 with a target mass of 50,000 and mass tolerance of 20 ppm. Charge states between 3 and 100 were included, and the Intact Protein peak model was selected. Further data analysis was performed using in-house Python scripts.

### CE-SDS

AAV samples with a titer in the range of 2 × 10^12^ to 1 × 10^13^ vg/mL in 15 μL were mixed with 1.8 μL of 150 mM N-ethylmaleimide in 4% SDS and incubated at 70°C in a heating block for 5 min. Afterward, the reaction mixture was spun down and cooled for 10 min; 2.25 μL of 2.5 mM FQ Dye reagent (3-(2-furoyl)quinoline-2-carboxaldehyde in DMSO) and 1.5 μL of 30 mM potassium cyanide were added and the reaction mixture was incubated for 10 min at 70°C. The reaction was stopped by addition of 42 μL of 1% SDS solution with subsequent incubation at 70°C for 5 min. Samples were cooled and spun down, 30 μL water was added. A final centrifugation step for 2 min at 4,000 rpm was done before they were injected in a PA 800 Plus Pharmaceutical Analysis System (Sciex) with 5.0 kV for 6 s and separation at 15.0 kV for 30 min. The detection was carried out with a laser-induced fluorescence detector at an excitation wavelength of 488 nm and an emission bandpass filter of 600 nm (dynamic range: 100 RFU; peak width: 16–25 nm).

## Data Availability

All data related to the work presented here are available through the public depository MassIVE: MSV000090582, ftp://massive.ucsd.edu/MSV000090582/.

## Supplemental information

Document S1. Figures S1–S5 and Tables S1–S4

Document S2. Article plus supplemental information
