# Supplementary material for: Assessing production variability in empty and filled adeno-associated viruses by single molecule mass analyses
Source: Mol Ther Methods Clin Dev. 2022 Nov 15;27:491–501. doi: 10.1016/j.omtm.2022.11.003 (PMC9706604; doi:10.1016/j.omtm.2022.11.003)
Supplement: Document S1. Figures S1–S5 and Tables S1–S4 [file mmc1.pdf]

OMTM, Volume *27*

Supplemental information

Assessing production variability in empty and filled adeno-associated viruses by single molecule mass analyses

Eduard H.T.M. Ebberink, Alisa Ruisinger, Markus Nuebel, Marco Thomann, and Albert J.R. Heck

Table S1: Theoretical genome sizes and masses of incorporated CMV-GFP genes. Approximate masses were calculated based on the amount of nucleotides in the transgenes according to the following formula: *mass ssDNA = (# nucleotides x 303.7) + 79.0*.

	**Genome size**	
	**Nucleotides**	**Mass (MDa)**
**Vir CMV-GFP**	2555	0.788
**Vig CMV-eGFP**	2433	0.751
**Sir CMV-eGFP**	3345	1.030

Table S2: Delta masses observed between the different subspecies found in the CMV-GFP packaged AAVs (see Figures S3 and Table 1). The fitted means of various subspecies were subtracted from each other to approximate the level of ssDNA encapsidation and compare delta masses between MP and CDMS.

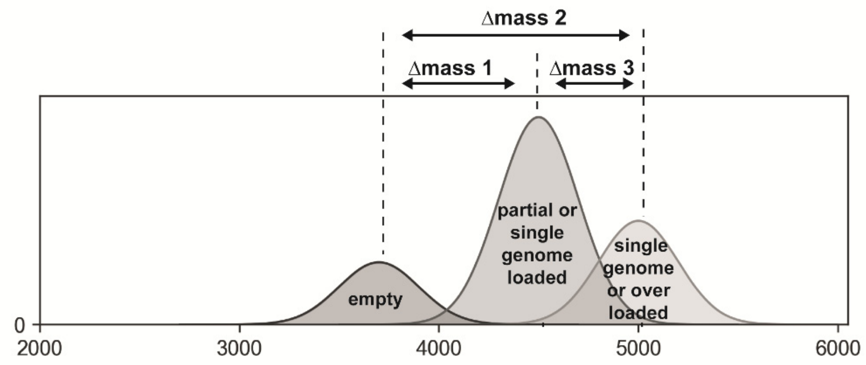

	∆mass 1 (MDa)		∆mass 2 (MDa)		∆mass 3 (MDa)	
	**MP**	**CDMS**	**MP**	**CDMS**	**MP**	**CDMS**
**AAV8_Vir_GFP**	0.73 ± 0.03	0.73 ± 0.02	1.28 ± 0.03	1.29 ± 0.02	0.55 ± 0.04	0.56 ± 0.01
**AAV8_Sir_GFP**	-	-	1.16 ± 0.05	1.17 ± 0.03	-	-
**AAV8_Vig_GFP**[a]	0.89 ± 0.07	0.79 ± 0.01	1.38 ± 0.06	1.27 ± 0.02	0.49 ± 0.08	0.48 ± 0.02
**AAV2_Vir_GFP**[a]	0.66 ± 0.07	0.78 ± 0.04	1.23 ± 0.08	1.41 ± 0.04	0.57 ± 0.11	0.63 ± 0.06
**AAV2_Sir_GFP**	0.66 ± 0.08	0.60 ± 0.03	1.19 ± 0.09	1.15 ± 0.03	0.53 ± 0.09	0.55 ± 0.03
**AAV2_Vig_GFP**	0.72 ± 0.09	0.74 ± 0.01	-	-	-	-

[a]*All values were taken from Table 1 CMV-GFP packaged AAVs, except for AAV8_Vig_GFP and AAV2_Vir_GFP. These two samples do not contain empty capsids, instead the fitted means of respectively AAV8_Vig and AAV2_Vir were used. Values in this table represent the subtracted mass ± standard deviation.*

Table S3: Mass assignments, annotation, and relative quantification of all detected proteoforms following LC-MS of empty AAV8 samples from the three different suppliers (see Figure 4).

	PTM	Found mass (Da)	Theoretical mass (Da)	Relative abundance (%)
Virovek AAV8				
VP1(2-738)	+Ac	81666.8	81667.3	90.3
	+Ac +P	81747.9	81747.3	100
	+Ac +2P	81828.1	81827.3	7.0
VP2(139-738)		66519.2	66518.6	53.0
	+P	66598.8	66598.6	100
	+2P	66682.4	66678.6	2.6
VP3(205-738)		59762.8	59763.1	26.6
	+Ac	59805.2	59805.1	100
VP3 variant (213-738)	+Ac	59192.1	59192.4	3.7
Sirion AAV8				
VP1(2-738)	+Ac	81666.7	81667.3	100
	+Ac +P	81747.3	81747.3	38.2
VP2(139-738)	-AlaPro	66350.1	66350.4	7.6
	-AlaPro+P	66428.7	66430.4	2.6
		66517.9	66518.6	100
	+P	66598.3	66598.6	75.3
	+2P	66680.0	66678.6	2.7
VP3(205-738)	+Ac	59805.0	59805.1	100
VP3 variant (213-738)	+Ac	59192.1	59192.4	7.2
Vigene AAV8				
VP1(2-738)	+Ac	81666.6	81667.3	100
	+Ac +P	81747.7	81747.3	26.7
VP2(139-738)	-AlaPro	66349.9	66350.4	4.0
		66518.6	66518.6	100
	+P	66598.5	66598.6	33.4
	*	66691.0	-	38.5
	* +P	66771.5	66771.0	11.0
VP3(205-738)	+Ac	59805.4	59805.1	100
VP3 variant (213-738)	+Ac	59192.5	59192.4	4.6

Table S4: Mass assignments, annotation, and relative quantification of all detected proteoforms following LC-MS of empty AAV2 samples from the three different suppliers (see Figure 4).

	PTM	Found mass (Da)	Theoretical mass (Da)	Relative abundance (%)
Virovek AAV2				
VP1(2-735)		81856.4	81855.5	100
	+P	81938.4	81935.5	8.0
VP2(139-735)		66488.7	66488.4	100
	+P	66568.3	66568.4	8.0
VP3(204-735)	+Ac	59974.3	59974.2	100
VP3 variant (212-735)	+Ac	59301.2	59301.4	2.2
Sirion AAV2				
VP1(2-735)		81859.9	81855.5	100
	+P	81943.6	81935.5	9.9
VP2(139-735)		66488.2	66488.4	100
	+P	66573.0	66568.4	2.0
VP3(204-735)	+Ac	59974.0	59974.2	100
VP3 variant (212-735)	+Ac	59301.6	59301.4	14.3
Vigene AAV2				
VP1(2-735)		81855.5	81855.5	100
VP2(139-735)	-AlaPro	66320.3	66320.2	1.2
		66488.7	66488.4	100
	*	66661.6	-	53.6
VP3(204-735)	+Ac	59974.4	59974.2	100
VP3 variant (212-735)	+Ac	59301.6	59301.4	5.7

Figure S1

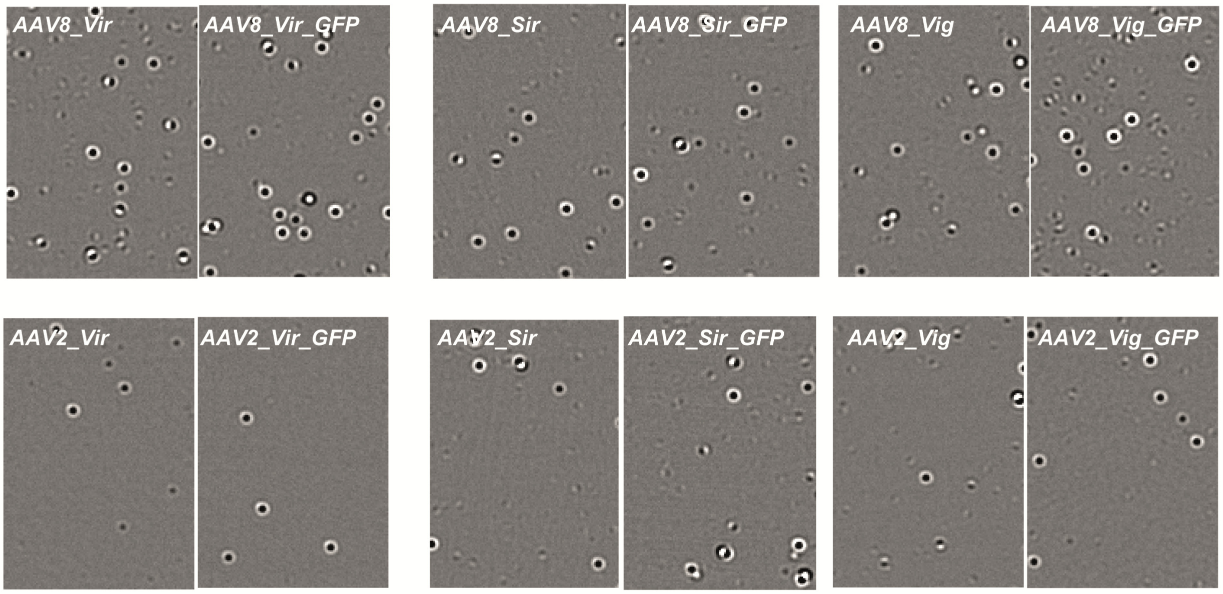

Figure S1: Scattering events of AAVs measured by MP. For each AAV sample a frame is given in which the landing of AAVs on the glass coverslip and subsequent scattering effect can be seen.

Figure S2

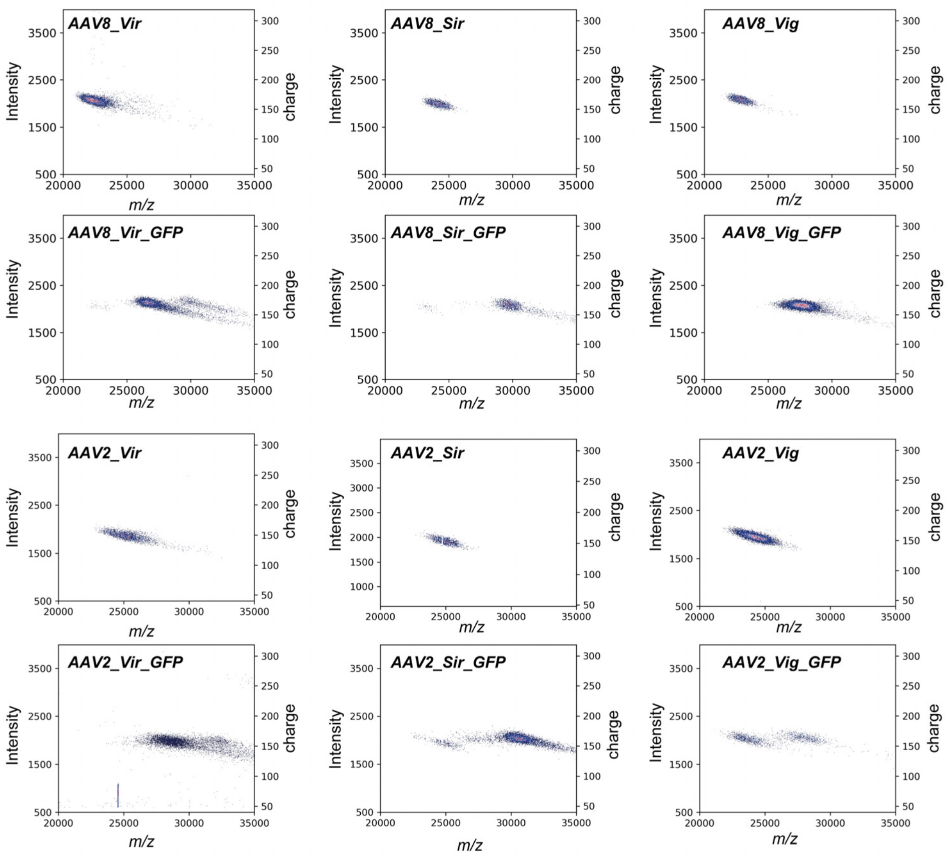

Figure S2: 2D-histograms of *m/z* versus charge. Displayed are the 2D histograms of CDMS measurements given in Figure 2 and 3. Bin widths of 25 Th and 10 arbitrary units for respectively *m/z* and intensity. The charges are extracted from the intensity values. The color code represents the amount of particles ranging from blue to red for respectively low and high values.

Figure S3

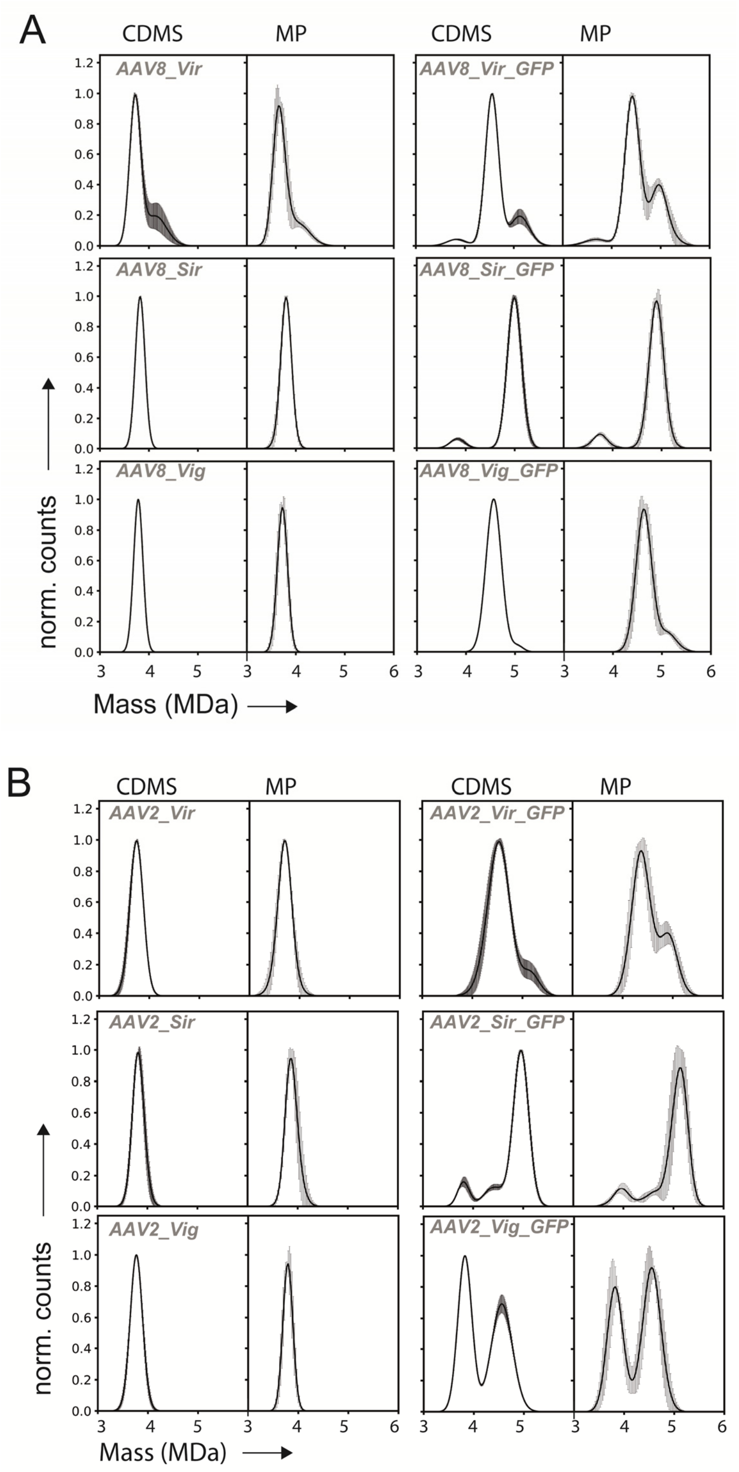

Figure S3: Fitted AAV mass distributions of multiple CDMS and MP measurements. Following at least 3 repeats of the CDMS and MP assays, Gaussian fits were averaged and normalized to the highest value. Displayed are the average fits of **A**) AAV8 and **B**) AAV2 repeats. The error bar represent the standard deviation in normalized counts within each bin.

Figure S4

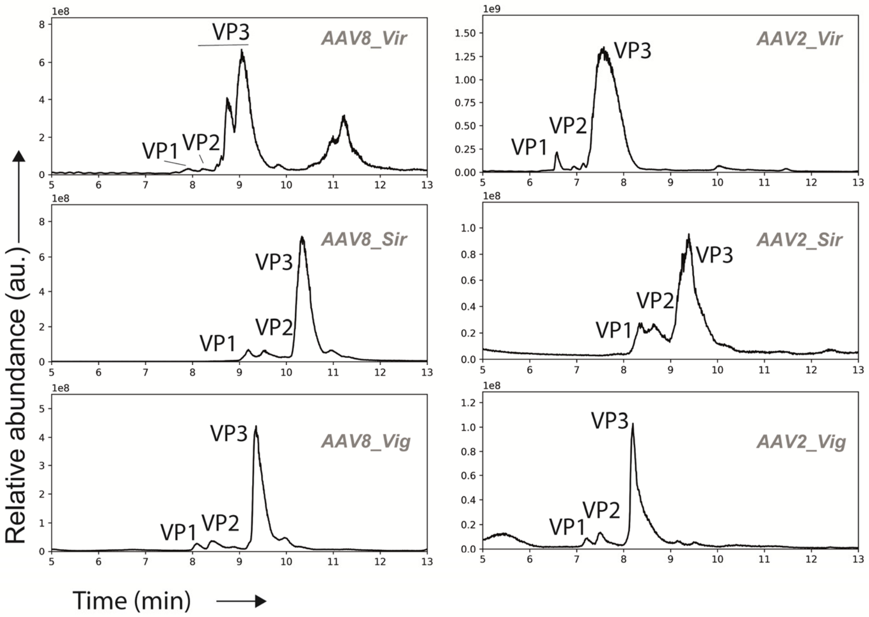

Figure S4: LC-MS traces of the total ion current. Displayed are chromatograms of the total ion current taken from the LC-MS measurements of Figure 4.

Figure S5

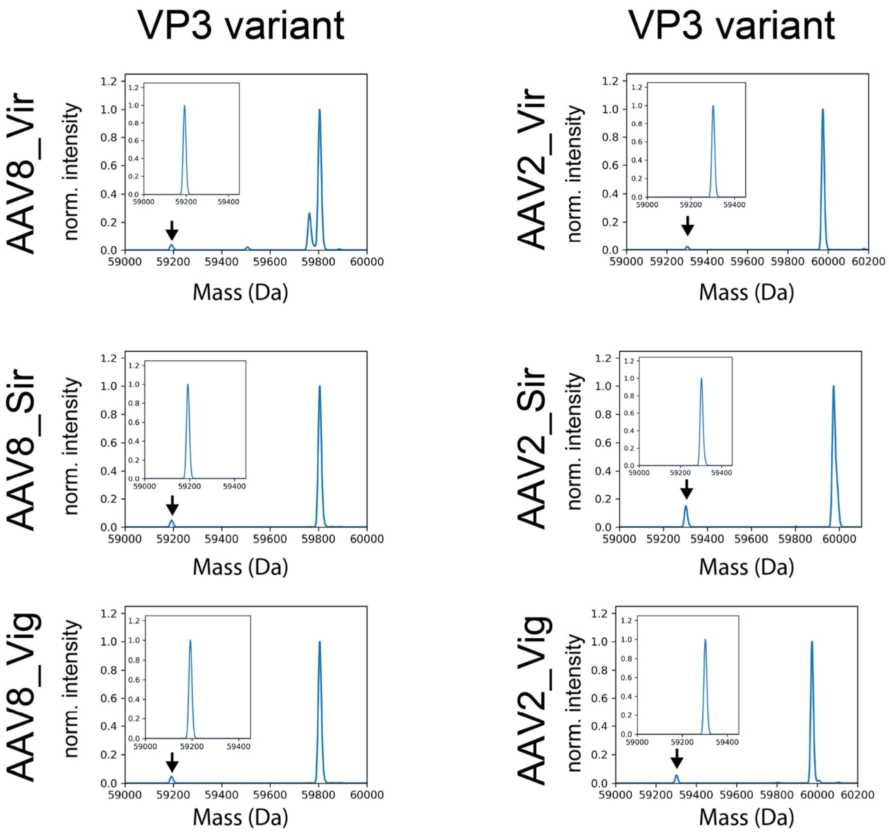

Figure S5: LC-MS profile of VP3 variants. Displayed are the deconvoluted masses of VP3 variants that have the first N-terminal residue at Ala212 for AAV2 and Ala213 for AAV8, following expression initiated at Met211 or Met212 respectively. The variant is indicated with an arrow. The inset displays a close up of the deconvoluted peak.
